# Non-contrast myocardial perfusion in rest and exercise stress using systolic flow-sensitive alternating inversion recovery

**DOI:** 10.1007/s10334-021-00992-3

**Published:** 2021-12-27

**Authors:** Markus Henningsson, Carl-Johan Carlhäll, Tino Ebbers, Johan Kihlberg

**Affiliations:** 1 Unit of Cardiovascular Sciences, Department of Health, Medicine and Caring Sciences, Linköping University, Linköping, Sweden; 2 Center for Medical Image Science and Visualization (CMIV), Linköping University, Linköping, Sweden; 3 Department of Clinical Physiology in Linköping, Department of Health, Medicine and Caring Sciences, Linköping University, Linköping, Sweden; 4 Department of Radiology, Department of Health, Medicine and Caring Sciences, Linköping University, Linköping, Sweden

**Keywords:** Non-contrast myocardial perfusion, Exercise stress test, Systolic flow-sensitive alternating inversion recovery, Arterial spin labeling

## Abstract

**Objective:**

To evaluate systolic flow-sensitive alternating inversion recovery (FAIR) during rest and exercise stress using 2RR (two cardiac cycles) or 1RR intervals between inversion pulse and imaging.

**Materials and methods:**

1RR and 2RR FAIR was implemented on a 3T scanner. Ten healthy subjects were scanned during rest and stress. Stress was performed using an in-bore ergometer. Heart rate, mean myocardial blood flow (MBF) and temporal signal-to-noise ratio (TSNR) were compared using paired *t* tests.

**Results:**

Mean heart rate during stress was higher than rest for 1RR FAIR (85.8 ± 13.7 bpm vs 63.3 ± 11.1 bpm; *p* < 0.01) and 2RR FAIR (83.8 ± 14.2 bpm vs 63.1 ± 10.6 bpm; *p* < 0.01). Mean stress MBF was higher than rest for 1RR FAIR (2.97 ± 0.76 ml/g/min vs 1.43 ± 0.6 ml/g/min; *p* < 0.01) and 2RR FAIR (2.8 ± 0.96 ml/g/min vs 1.22 ± 0.59 ml/g/min; *p* < 0.01). Resting mean MBF was higher for 1RR FAIR than 2RR FAIR (*p* < 0.05), but not during stress. TSNR was lower for stress compared to rest for 1RR FAIR (4.52 ± 2.54 vs 10.12 ± 3.69; *p* < 0.01) and 2RR FAIR (7.36 ± 3.78 vs 12.41 ± 5.12; *p* < 0.01). 2RR FAIR TSNR was higher than 1RR FAIR for rest (*p* < 0.05) and stress (*p* < 0.001).

**Discussion:**

We have demonstrated feasibility of systolic FAIR in rest and exercise stress. 2RR delay systolic FAIR enables non-contrast perfusion assessment during stress with relatively high TSNR.

## Introduction

Cardiovascular magnetic resonance (CMR) allows non-invasive assessment of myocardial ischemia using contrast-enhanced first-pass myocardial perfusion [1]. Although this technique has excellent diagnostic and prognostic performance [2], cautious use of gadolinium-based contrast agent is recommended in patients with poor renal function and the risk of gadolinium accumulation in body and brain are limitations [3]. Furthermore, accurate and precise perfusion quantification is challenging using contrast-enhanced CMR which primarily remains a qualitative technique [4].

In recent years, there has been increased interest in developing CMR techniques to assess myocardial ischemia without the use of contrast agents or pharmacological stress agents. This includes T1 mapping [5, 6], oxygen sensitive (T2* or T2 weighted) imaging [7] and arterial spin labeling (ASL) [8]. Of these, ASL is the most comparable to conventional contrast-enhanced first pass perfusion as it aims to quantify blood flow in the myocardial microvasculature. The most commonly used and well-validated ASL method for myocardial perfusion is the flow-sensitive alternating inversion recovery (FAIR) technique [9–11]. Studies have been performed using myocardial FAIR during rest and exercise in healthy subjects and patients with myocardial ischemia, demonstrating the feasibility of this approach to estimate myocardial blood flow (MBF) reserve [12–14].

A limitation of ASL in general, and myocardial FAIR in particular, is the low signal sensitivity of this technique. This is primarily due to the intrinsically low signal variability which may be attributed to perfusion of maximum 4–8% [8, 15], approximately the volume of microvascular blood in healthy myocardial tissue [16]. Furthermore, FAIR relies on image subtraction of a tagged and control image, each acquired using selective and non-selective inversion recovery, respectively. As a result, FAIR images have a low signal-to-noise ratio (SNR) and are susceptible to spatial misregistration caused by cardiac and respiratory motion. To mitigate these challenges, multiple averages are typically acquired, at least 6 for each slice [17]. Other measures which have been employed to increase the SNR include the use of 3 T scanners with balanced steady-state free precession readouts [18], respiratory motion compensation [19], and different ASL techniques [20, 21]. Temporal signal to noise (TSNR) may also suffer during physiological stress, when the heart rate increases and the duration between inversion pulse and imaging becomes short. Waiting multiple cardiac cycles between inversion and imaging can mitigate this at the cost of slightly extended scan time and may be a practical approach to increase TSNR during stress [22]. Typically, the temporal distance between the inversion pulse and image acquisition is one cardiac cycle. However, for high heart rates this may be very short which results in a lower amount of inflowing blood in the labeled image slice, reducing the perfusion SNR. A previous study using cardiac FAIR by Javed et al. [23], investigated using two cardiac cycles delay between inversion and imaging instead of one in four healthy volunteers. However, no systematic study has been performed to assess the merits of a two cardiac cycle delay approach for cardiac FAIR during rest and stress.

Another approach to increase robustness of cardiac FAIR is to time the data acquisition to the systolic rather than diastolic rest period, as this leads to an increased area of analyzable myocardium and may be preferable during high heart rates as typically encountered in stress perfusion [24–26]. However, with conventional image acceleration systolic FAIR suffers from increased physiological noise. Even though advanced image acceleration techniques such as compressed sensing allow reducing the acquisition time to approximate the shorter systolic rest period, so far, systolic FAIR has only been demonstrated during rest [27]. The objective of this study was to evaluate systolic FAIR during rest and exercise stress in 10 healthy subjects. Furthermore, we explore the use of waiting 2RR intervals between inversion pulse and imaging, and compare it to the conventional 1RR delay in rest and stress conditions.

## Materials and methods

All experiments were performed on a 3 T Philips scanner (Philips Healthcare, Best, The Netherlands) using a 24-channel torso coil. The study was approved by the institutional review board (Dnr 2016/546-32) and all subjects provided written informed consent.

**Fig. 1:**
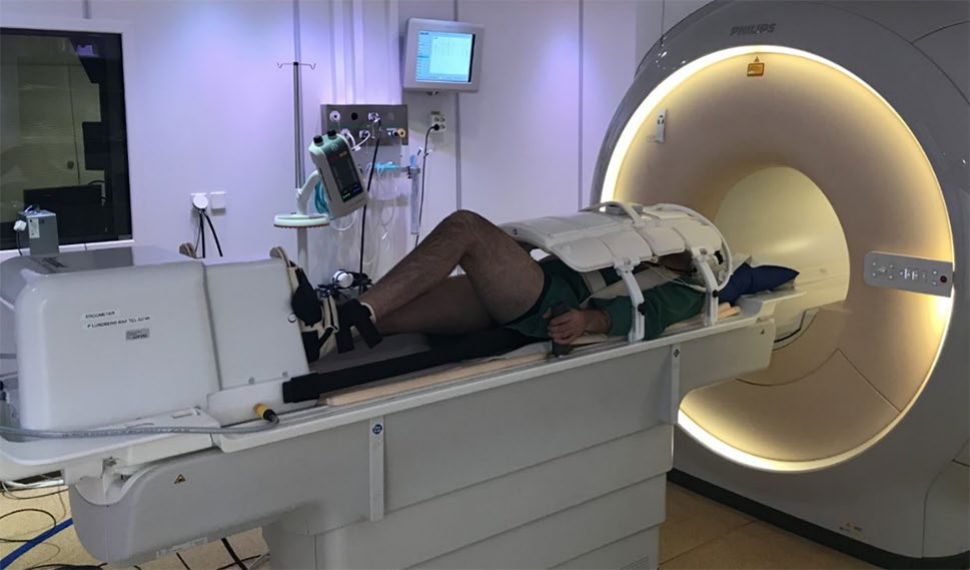
Step ergometer used for the exercise test. Exercise was performed in the scanner bore between breath holds when stress images were acquired

Ten healthy volunteers (age: 32.6 ± 4.5 years; 5 male) were scanned at rest and during exercise stress. For the exercise stress test an in-bore ergometer (Ergospect Gmbh, Innsbruck, Austria), shown in Fig. 1, with step function was used. Exercise was performed in the scanner bore for approximately 2 min before image acquisition was started to find a stable exercise level which could be tolerated for 5 min while also ensuring that the heart rate was elevated compared to rest. Exercise was briefly paused during the approximately 10 s breath-hold FAIR acquisitions to stabilize the ECG signal and ensure reliable triggering was obtained. Exercise was resumed again during the 20–30 s waiting periods between breath holds.

The prosed systolic FAIR technique was ECG-triggered and used double-gating to ensure the inversion pulses were performed in the same cardiac phase but a preceding cardiac cycle as the image acquisition. In this work, cardiac FAIR was performed either with one cardiac cycle delay between inversion pulse and imaging (1RR FAIR) or with two cardiac cycle delay (2RR FAIR). Apart from this modification, the double-gated myocardial FAIR acquisition was performed as in previous studies, where tagged and control images were acquired within one breath hold of approximately ten seconds with 8 s of delay between inversion pulses. In addition, an M_0_ image was acquired in a separate scan without any magnetization preparation to estimate baseline magnetization for every data set. Myocardial blood flow (MBF) was calculated for both 1RR FAIR and 2RR FAIR using the formula for double-gated myocardial ASL:

1 $${\text{MBF}} = \frac{1}{{2M_{0} }}\left( {\frac{C}{{TI_{C} \cdot e^{{\frac{{ - TI_{C} }}{{T_{1} }}}} }} - \frac{T}{{TI_{T} \cdot e^{{\frac{{ - TI_{T} }}{{T_{1} }}}} }}} \right),$$

where *C* and *T* are the control and tagged images, respectively. T_1_ is the longitudinal relaxation time for blood at 3 T, estimated at 1700 ms [28].

**Fig. 2:**
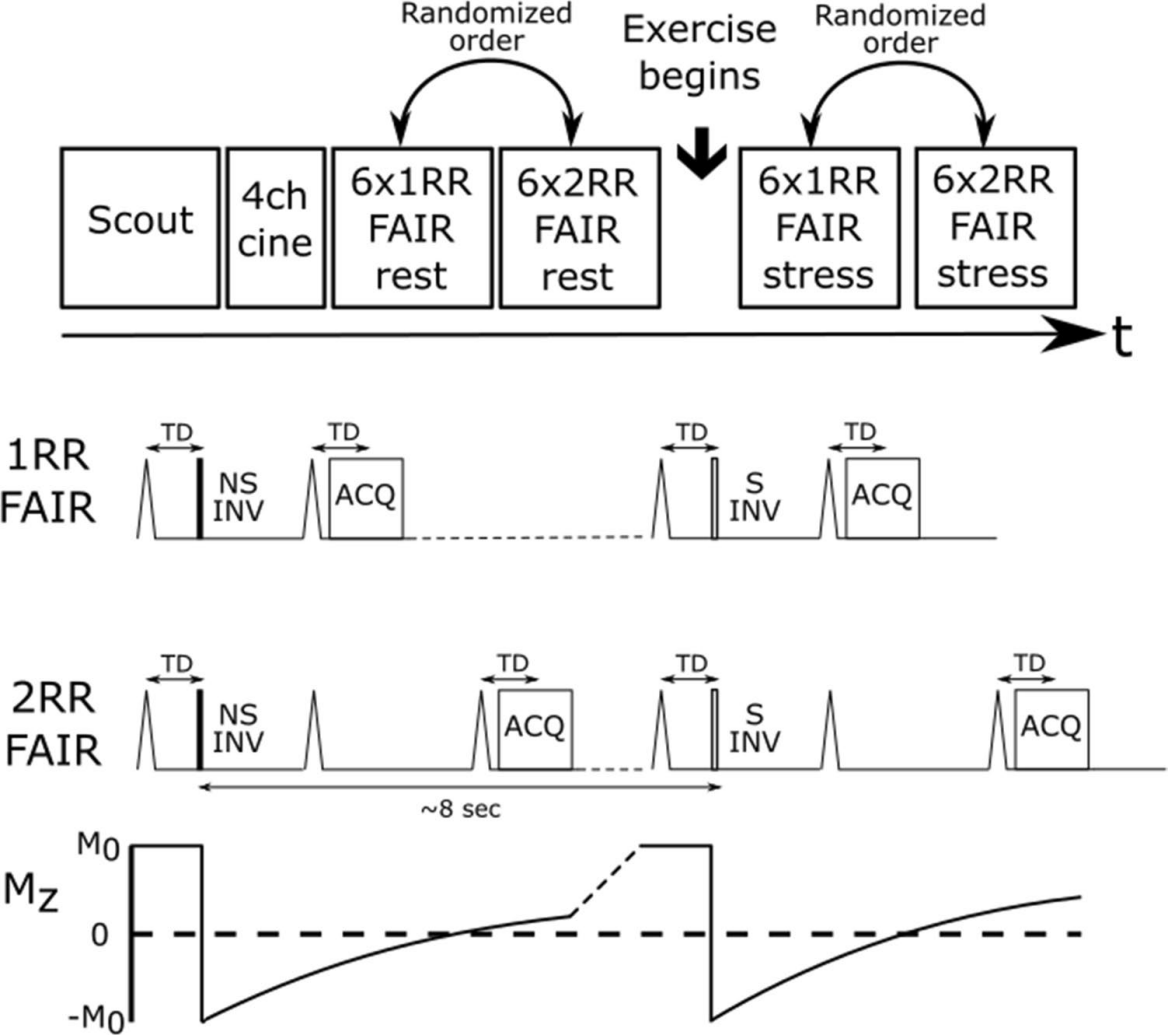
Illustration of the scan protocol for the rest and stress experiments, including scouts to localize the short axis and 4 chamber views, 4 chamber (4ch) cine to determine the end-systolic trigger delay (TD), and rest and stress 1RR and 2RR FAIR with 6 averages each. The order of the FAIR scans was randomized for the different subjects. Pulse sequence diagrams for the 1RR and 2RR FAIR are also shown, with non-selective inversion pulses (NS INV) and selective inversion pulses (S INV) with the same TD, but preceding cardiac cycles, as the image acquisition (ACQ). The inversion pulses were spaced approximately 8 s apart to allow for near complete (> 99%) M_z_ recovery

For the in-vivo experiments, a mid-ventricular 2D FAIR image was acquired in short-axis and each data set consisted of six pairs of tagged and control images, each pair acquired during a breath-hold and with alternating order of the control/tagged images. Imaging parameters were: spatial resolution = 2 × 2 mm^2^, FOV = 300 × 300 × 10 mm, flip angle = 50°, TR/TE = 2.2/1.1 ms, acquisition time = 110 ms, 25 ramp-up radiofrequency pulses preceded the data acquisition with linearly increasing flip angles, from 1 to 49º with 2 incrementsº. Compressed SENSitivity Encoding (SENSE) was used for image acceleration with a factor of 3. The systolic rest period was visually determined using a four-chamber cine slice with 50 cardiac phases acquired during rest and assumed to be the same for the stress scans [29]. The scan protocol and 1RR and 2RR FAIR pulse sequences are shown in Fig. 2.

### Data analysis and statistics

The images were processed offline to generate pixelwise maps of MBF. First, all images (tagged, control and M_0_) were jointly co-registered using non-linear image registration to account for differences in respiratory position within and between breath-holds [30]. MBF maps were then calculated for each of the six pairs of control and tagged images, as previously outlined for double-gated myocardial FAIR [11]. The final MBF maps were then generated by averaging across the six MBF that were calculated for each control and tagged pair.

From the acquired images MBF was estimated and temporal signal-to-noise ratio (TSNR) was calculated based on a region of interest which was manually drawn to include the entire myocardium in the mid-ventricular slice. TSNR was defined as the ratio between the mean MBF and the standard deviation of the MBF across the 6 measurements [13].

Two-tailed paired student’s *t* test were used to statistically compare group mean differences for heart rate, mean MBF and TSNR. A threshold of *P* < 0.05 was used to define statistically significant differences.

## Results

The rest and stress 1RR FAIR and 2RR FAIR scans were successfully performed in all but one volunteer, where excessive respiratory motion during the 1RR FAIR stress scan could not be sufficiently corrected and resulted in uninterpretable MBF maps. Therefore, 1RR and 2RR FAIR data for this volunteer were not included in the statistical analysis to allow pairwise comparisons.

**Fig. 3:**
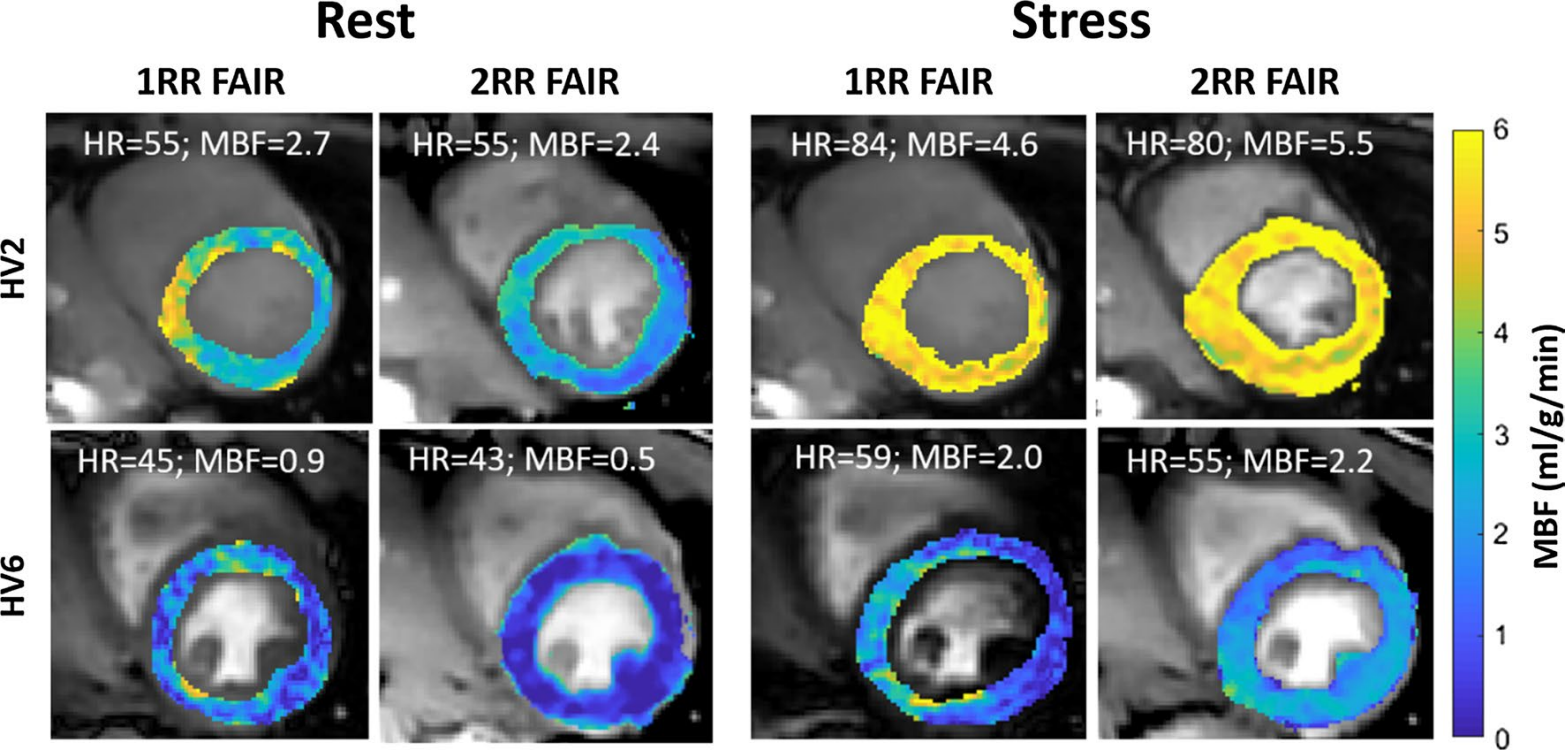
Example perfusion images for two healthy volunteers (HV2 and 6) during rest and stress using 1RR and 2RR FAIR. Increased heart rate and myocardial blood flow (MBF) is observed during the stress test compared to rest

**Fig. 4:**
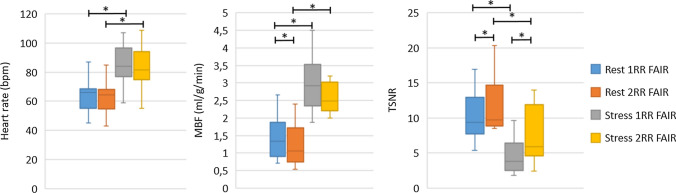
Boxplots of mean heart rate, myocardial blood flow (MBF) and temporal signal-to-noise ratio (TSNR) for 1RR and 2RR FAIR during rest and stress. Statistical differences (*p* < 0.05) are indicated by asterisk

**Fig. 5:**
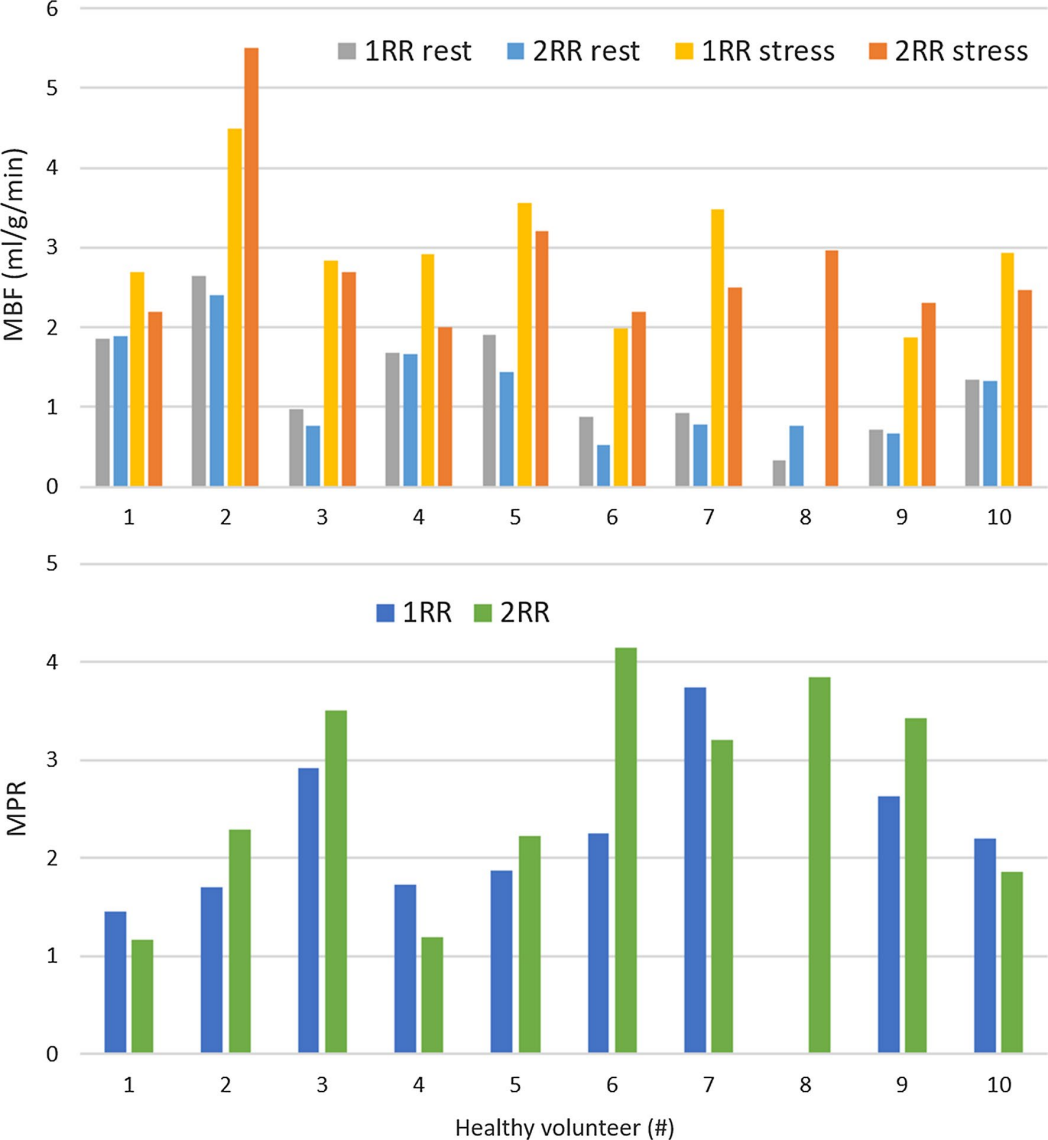
Mean myocardial blood flow (MBF) for all 10 healthy subjects during rest and stress and for 1RR and 2RR FAIR (top graph). 1RR FAIR stress images for healthy subject 8 was excluded due to significant artifacts. Mean myocardial perfusion reserve (MPR) for all 10 volunteers for 1RR and 2RR FAIR are shown in the bottom graph

Example perfusion maps for two subjects using 1RR and 2RR FAIR during rest and stress are shown in Fig. 3 with markedly increased perfusion during stress compared to rest. Figure 4 shows the group average heart rate, MBF and TSNR during rest and stress for both 1RR FAIR and 2RR FAIR. The mean ± standard deviation end-systolic trigger delay was 319 ± 36 ms. The mean heart rate during the stress scan was significantly higher compared to rest for both 1RR FAIR (85.8 ± 13.7 bpm vs 63.3 ± 11.1 bpm; *p* < 0.01) and 2RR FAIR (83.8 ± 14.2 bpm vs 63.1 ± 10.6 bpm; *p* < 0.01). However, there was no statistical difference in heart-rate between 1 and 2RR FAIR for either rest and stress. The MBF of the stress scans were significantly higher compared to the rest scans for both 1RR FAIR (2.97 ± 0.76 ml/g/min vs 1.43 ± 0.6 ml/g/min; *p* < 0.01) and 2RR FAIR (2.8 ± 0.96 ml/g/min vs 1.22 ± 0.59 ml/g/min; *p* < 0.01). The MBF was significantly higher using 1RR FAIR during rest compared to 2RR FAIR (*p* < 0.05), but not during stress. The myocardial perfusion reserve (MPR) was calculated for all volunteers using 1RR FAIR and 2RR FAIR, where both rest and stress were available. The MPR for 1RR FAIR was 2.28 ± 0.72 and for 2RR FAIR 2.56 ± 1.07, which was not statistically significant (*p* = 0.32). The TSNR was significantly lower for the stress scans compared to rest for both 1RR FAIR (4.52 ± 2.54 vs 10.12 ± 3.69; *p* < 0.01) and 2RR FAIR (7.36 ± 3.78 vs 12.41 ± 5.12; *p* < 0.01). However, TSNR was significantly higher for 2RR FAIR compared to 1RR FAIR for both rest (*p* < 0.05) and stress scans (*p* < 0.001). The measured MBF and MPR for each volunteer using 1RR FAIR and 2RR FAIR during rest and stress are shown in Fig. 5.

## Discussion

Here, we have demonstrated the ability to estimate perfusion during rest and exercise stress using systolic FAIR. We compared the use of 2RR interval delays between inversion pulse and imaging acquisition to 1RR delay and found that the former approach yielded higher TSNR at both rest and stress at the expense of producing, on average, 6% lower MBF values during rest.

Similar to previous studies exploring the use of 1RR and 2RR FAIR techniques, we found a reduction in MBF for the 2RR approach [23]. This may be explained by the partial labeling of the inflowing blood for 2RR but not 1RR FAIR. Despite the loss of MBF accuracy it can be advantageous to use a 2RR delay as it appears to increase the TSNR, which is particularly important during stress when physiological noise is higher. Although the lower MBF for 2RR compared to 1RR FAIR was significantly different only during rest, we hypothesize that any similar effect during stress may be masked by the increase in MBF variability during this scan. This variability may be attributed to differences in respiratory and cardiac motion between the 1RR and 2RR FAIR scans which are typically larger during rest than stress, but also actual differences in cardiac workload between the 1RR and 2RR scans.

The rest mean MBF was approximately 1.2–1.4 mL/g/min in this study. Others also reported a similar range (1.3–1.5 mL/g/min) [10, 13], but which is above the considered normal limit of 0.8 mL/g/min. The measured MBF was doubled during stress which is rather low considering the rule of thumb of 3.5 times increase [31]. We observed only a modest heart rate increase in the scanned healthy subjects during exercise stress, which may indicate that the work load was relatively low, yielding a similarly moderate perfusion increase. Another source of reduced difference between rest and stress perfusion could be that the subjects were slightly anxious in rest. However, physiological stress derived from muscle work is not the same as pharmacological stress. Furthermore, no correction was made for how well-trained the people were, and fitness status may explain differences and the variability in physiological response to stress. The reduction in TSNR during stress compared to rest may be attributed to a combination of changes in the systolic rest period relative to rest (which would increase physiological noise) and actual changes in perfusion across the 6 breath-holds. Further work is required to limit the adverse effects of physiological noise during stress, including improved respiratory motion compensation, such as prospective correction or breathing guidance. Physiological noise due to cardiac motion could be further reduced by shortening the acquisition window using alternative acceleration techniques. Finally, the use of pharmacological stress is likely to be extremely beneficial for reducing respiratory and cardiac motion (mainly due to ECG-mistriggering cause by the exercise) and will be explored in future patient studies. Nevertheless, the proposed systolic FAIR technique appears robust and may offer an attractive approach for myocardial perfusion assessment during stress without the use of contrast agents or pharmacological stress.

A clinical exercise test aims to reach an age-based heart rate, to induce ischemia which may be hidden at rest [32]. In this study, a modest significant heart rate increase of 35% was achieved, which for patients might not be sufficient given the low average age of the subjects. However, similar experiments have been performed to investigate whether a difference in perfusion could be detected using hand grip exercise [13]. The scanning protocol and equipment in this study was designed to evaluate a particular cardiac ASL technique in healthy subjects. To develop a clinical protocol requires either pharmacological stress, where drugs are given strictly according to weight and tolerance or exercise with an age-based target heart rate [32], and including at least three short-axis views of the heart [4].

The clinically most common modality for perfusion measurement is Single Photon Emission Computed Tomography (SPECT), but it provides low temporal and spatial resolution. Photon Emission Tomography (PET) is considered the clinical gold standard but requires the use of ionizing radiation, unlike MRI [33]. There are various MRI techniques used clinically to measure perfusion in the brain without Gd contrast, some of which have been implemented for cardiac MRI [8]. In particular, ASL, blood oxygenation level dependent sequence (BOLD) [7], intravoxel incoherent motion (IVIM) [34] and T_1_ mapping [35] have been evaluated in animal experiments, healthy subjects and patients. ASL is the most widely used non-contrast media MRI technology in the brain due to its robustness [36] and, therefore, probably has the greatest chance of also succeeding in the heart.

The study has several limitations: The study comprised of a small number of healthy subjects, and larger studies including patients with coronary artery disease are warranted to evaluate this technique, including validation against quantitative reference techniques, such as contrast-enhanced MRI. Furthermore, validation using a perfusion phantom would be desirable and will be the focus of future work [37, 38]. The use of a step ergometer to stress the myocardium has practical challenges, including a likely higher incidence of large-scale bulk respiratory and cardiac motion compared to conventional pharmacological stress, where patients can lie still. Furthermore, the step ergometer requires additional patient preparation to ensure the equipment is tightly fastened to the patient which increases scan complexity and adds examination time overhead. However, it can be desirable to avoid the use of pharmacological stress agents, and alternative exercise approached may be explored in those patients [39]. A technical limitation of this technique is the requirement for relatively long breath-holds which can be challenging to consistently maintain during stress, and particularly for patients with cardiovascular disease. Respiratory-induced motion in the through-plane direction may cause MBF quantification errors if it occurs between the slice-selective inversion and image acquisition, which may be particularly problematic for the 2RR FAIR technique, where this delay is the longest. Prospective motion correction can mitigate these limitations, and techniques to enable motion-tolerant free-breathing FAIR would be desirable to facilitate patient scans and clinical translation [19, 40, 41]. A fixed T_1_ for blood of 1700 ms was used for all subjects. However, blood T_1_ varies particularly with hematocrit levels, and a subject-specific blood T_1_ measurement may yield more accurate MBF estimation. Finally, the systolic time was determined from cine images at rest, similar to a previous perfusion publication [26], but no control cine was performed in stress. However, the systolic rest perdiod is invariant to heart rate changes compared to the diastolic rest period (29).

## Conclusions

We have demonstrated the feasibility of systolic FAIR during rest and exercise stress. Systolic FAIR with 2RR delay between inversion and imaging enables non-contrast perfusion assessment during stress with relatively high TSNR. MBF may be slightly underestimated compared to 1RR FAIR due to partial signal saturation. Further studies are warranted to investigate the diagnostic potential of this technique in patients with coronary artery disease.
